# Matched linac stereotactic radiotherapy: An assessment of delivery similarity and distributive patient‐specific quality assurance feasibility

**DOI:** 10.1002/acm2.13652

**Published:** 2022-05-15

**Authors:** Simon K Goodall, Leon Dunn, Jonathan Dunning, Luis Muñoz, Pejman Rowshanfarzad, Martin A Ebert

**Affiliations:** ^1^ School of Physics, Mathematics, and Computing, Faculty of Engineering and Mathematical Sciences University of Western Australia Crawley Western Australia Australia; ^2^ GenesisCare Wembley Western Australia Australia; ^3^ GenesisCare Fitzroy Victoria Australia; ^4^ GenesisCare Bedford Park South Australia Australia; ^5^ Centre for Medical Radiation Physics University of Wollongong New South Wales Australia; ^6^ Department of Radiation Oncology Sir Charles Gardiner Hospital Nedlands Western Australia Australia; ^7^ 5D Clinics Perth Western Australia Australia

**Keywords:** beam‐matching, distributive PSQA, match, SBRT, VMAT

## Abstract

Matching multiple linacs to common baseline data allows patients to be treated, and patient‐specific quality assurance (PSQA) to be completed on any linac. Stereotactic body radiotherapy (SBRT) requires higher levels of accuracy and quality assurance than routine radiotherapy. The achieved linac matching must therefore be evaluated before distributive treatment or PSQA models can be implemented safely. This investigation aimed to propose metrics for defining linacs to be matched for SBRT deliveries, assess 12 linacs against these criteria, and determine if a distributive PSQA model could be implemented by reviewing the rates of false PSQA results. Ten SBRT spine plans were delivered by 12 matched Elekta linacs and measured using one of seven SRS MapCHECK devices. For gamma criteria of (3%, 2 mm), 96.9% of equivalent location detectors, showed a range of gamma ≤ 1.0 and 99.9% showed a standard deviation of ≤ 0.5. For criteria of (3%,1 mm) and (2%,1 mm), these ranges decreased to 92.1% and 80.2% while the standard deviations decreased to 99.3% and 95.7%, respectively. The dose differences showed that 43.6%, 82.7%, and 91.4% of detectors had a dose range of ≤ 3.0%, ≤ 5.0%, and ≤ 6.0%, respectively. Standard deviations of dose differences were 1.5%, 2.5%, and 3.0% for 94.1%, 98.3%, and 99.5% of detectors, respectively. For the fleet of linacs, distributive PSQA yielded false results for 0.0%, 17.7%, and 33.0% of plans, equivalent to 1.2%, 3.5%, and 9.4% of detectors when using gamma criteria of (3%,2 mm), (3%,1 mm), or (2%,1 mm), respectively. These linacs could be considered matched for SBRT treatments and implement a distributive PSQA model when gamma analysis was completed with a criterion of (3%, 2 mm). For stricter criterion of (3%,1 mm) or (2%,1 mm), they did not meet the proposed metrics.

## 1 INTRODUCTION

The matching of multiple linear accelerators (linacs) within a single radiotherapy department increases the efficiency, flexibility, and redundancy available to the clinical team. When linacs have been shown to perform within a specified tolerance of a common set of baselines, patients can be treated on different linacs within a department on any given day in a distributive fashion. This allows greater flexibility in the event of linac downtime, scheduled or unexpected, and can eliminate the risks associated with the need to treat a patient on one specific linac.^1^, ^2^, ^3^

By extension, patient‐specific quality assurance (PSQA) could also be completed on any linac. Such “distributive PSQA” could be particularly beneficial to multicenter departments which may have remote sites with limited staff availability, or high workload centers with limited access to specific linacs. Distributive PSQA could also eliminate the requirement to own a large number of phantoms and detectors at every site, or to have the capacity to routinely transport equipment between sites as required, risking potential damage.

The ability to match linacs at a level sufficient for routine treatments and PSQA has been assessed in several studies.^1^, ^2^, ^3^, ^4^, ^5^, ^6^, ^7^, ^8^ Initial investigations focus on the ability to achieve a high level of matching between characteristics of the linac output, such as beam profiles, depth dose curves, and output factors.^1^, ^2^, ^4^ Further investigations consider the resultant similarity within composite dose deliveries for volumetric‐modulated arc therapy (VMAT) or intensity‐modulated radiotherapy (IMRT).^3^, ^5^, ^8^ More recently investigations have begun to consider the possibility of linac matching at the level required for stereotactic radiotherapy.^6^, ^7^

Stereotactic body radiotherapy (SBRT) is considered a high‐end treatment technique that requires a level of calibration accuracy and quality assurance above that required for routine radiotherapy.^9^, ^10^, ^11^ SBRT requires high spatial and dosimetric accuracy to ensure multiple small fields, delivered as highly‐modulated treatment plans, summate to create steep composite dose gradients. These increasingly steep dose gradients allow for the escalation of dose to targets in proximity to organs at risk (OAR), offering the potential for better clinical outcomes, but also more severe adverse side effects if the required accuracy is not achieved. SBRT spinal treatments are a key example, in which high doses are delivered to treatment volumes mere millimeters from the critical spinal cord.

There are currently few studies which consider the possibility of distributive patient treatments and PSQA for highly‐complex SBRT treatments at the composite dose delivery level.^12^, ^13^ Rijken et al. investigated distributive SBRT PSQA using nine matched linacs and two treatment plans, concluding the process feasible when using a gamma criterion of (3%, 2 mm).^12^ Larger variations in inter linac gamma results were however observed when using a stricter gamma criterion of (3%,1 mm) which may be more applicable to SBRT spine treatments when margins of 2 mm or less are often applied.^10^, ^14^, ^15^ Xu observed a high level of agreement between three matched Elekta linacs over a larger range of lung and brain plans but observed a different number of plans passing defined PSQA overall on each linac.^13^ The variation in PSQA results for a given plan was not presented; as such, the risk of a plan achieving a high gamma score on one linac yet a fail score on another cannot be evaluated. This inability to determine if a plan will pass or fail PSQA on an alternate linac is a major hurdle in the clinical realization of distributive SBRT treatments and PSQA, resulting in many of the benefits of linac matching being lost for the SBRT cohort of patients.

GenesisCare uses a single 6 MV flattening filter‐free (FFF) beam model and set of MLC modeling parameters within the Monaco treatment planning system (TPS; Elekta, Stockholm, Sweden) for a fleet of matched linacs across Australia. All Elekta linacs are matched to the criteria suggested by Rijken et al. during the commissioning process.^12^ The aims of this work were to

1. Evaluate the similarity of delivered, composite SBRT spine dose distributions, from linacs matched to a level above that recommended by the vendor, via comparison of PSQA gamma results;^12^
2. Investigate if the locations of disagreement between calculated and measured dose distributions were consistent across the fleet of linacs;
3. Determine the safety of a distributive PSQA program by evaluation of the frequency of false PSQA results observed.

## 2 METHODS

### 2.1 Treatment plan generation

A series of 10 VMAT SBRT spine treatment plans, previously treated within GenesisCare, was used throughout this investigation. During the clinical treatment process, five thoracic and five lumbar spine vertebra clinical target volumes (CTV) were generated across eight patients, each following the methods described by Cox et al.^14^ The details of each plan are given in Table 1, with plans containing an A or B indicating separate plans generated for different targets using the same CT set.

**TABLE 1:** Description of the key aspects of each treatment plan

Plan name	Vertebra	Arcs per beam	Number of beams	Arc length (^°^)	Fraction dose (cGy)	Total MU	MU/cGy
Plan1	T7	Single	2	360	900	4654	5.17
Plan2	T8	Dual	1	360	900	4643	5.16
Plan3	L1	Single	2	360	600	3135	5.23
Plan4‐A	L1	Dual	1	360	900	5333	5.93
Plan4‐B	T10	Dual	1	360	900	5289	5.88
Plan5	L4	Dual	1	360	800	3943	4.93
Plan6	T8	Dual	1	360	900	5440	6.04
Plan7‐A	T5	Dual	1	360	800	4683	5.85
Plan7‐B	L3	Dual	1	360	900	3565	3.96
Plan8	L4	Dual	1	360	900	6000	6.67

An MRI dataset had been obtained for each patient at the time of treatment and fused with the planning CT. The spinal cord was delineated by the treating radiation oncologist (RO) and grown isotropically by 2 mm to produce a planning risk volume (PRV). The treatment planning target volume (PTV) was generated as a 2 mm expansion of the CTV excluding the region incorporated by the spinal cord PRV. The treatment plan was optimized to achieve the prescription dose per fraction, as indicated in Table 1, to a minimum of 90% of the PTV while minimizing the spinal cord PRV dose. The plans were generated in Monaco 5.11.02 and calculated using a 1 mm isotropic dose grid and 1.0% statistical uncertainty per plan in line with work previously published.^16^ All treatment plans were calculated using the single 6 MV FFF beam model.

### 2.2 Linacs and SRS MapCHECK measurements

A total of 12 Elekta Versa HD linacs (Elekta, Stockholm, Sweden) were used within this study. These linacs were located across 11 clinical centers, over five different states of Australia. All treatment linacs had previously been matched to a single set of reference data using the criteria described by Rijken et al. and summarized in Table 2.^12^ Each had been commissioned in combination with the Monaco beam model for clinical use including SBRT treatments.

**TABLE 2:** Matching criteria used in this study, compared to the reference data

Characteristic	Tolerance
PDD_20,10_	0.5%
PDD points	1.0%
Profile points	1.0%
Output factors	1.0% (2.0% fields ≤ 1 × 1 cm^2^)
MLC Calibration	±0.5 mm

*Note*: Profile points are those within 80% of the field width.

Each treatment plan was copied onto a CT scan of a phantom housing the SRS MapCHECK (SRSMC) device (Sun Nuclear Corporation (SNC), Melbourne, FL) with the detector board aligned for a sagittal plane measurement. This orientation typically constitutes the steepest dose gradient region within an SBRT spine plan and encompasses the dose fall off from the PTV into the spinal cord PRV along the full superior to inferior range of the PTV. The PSQA plan calculation was also completed using an isotropic dose grid of 1 mm and a statistical uncertainty of 1.0% per plan. A total of seven SRSMC devices were used in this study, for each device the array and dose calibration processes described in the SNC manual were completed.^17^ Post‐calibration, a subset of the device performance checks described by Ahmed et al. were performed, including the array calibration check via device rotation.^18^ All results of these tests were in agreement with those observed by Ahmed et al. This device has previously proven to be capable of performing PSQA for a range of stereotactic treatment sites including spine.^19^

During the measurement process, the SRSMC was aligned to the MV isocenter. Each plan was delivered to the SRSMC and a single composite measurement was captured for the entire plan using the SNC Patient software version 8.4.1.2. This process was repeated for each linac, plan, and SRSMC combination used. A total of nine physicists completed measurements.

### 2.3 Similarity of deliveries

Gamma analysis of the measured and calculated dose distributions was completed while implementing the “calc shift” function within the SNC Patient software. This function applies small translational shifts to the calculated dataset to maximize the observed gamma score. This process ensured measurements made on each linac were not adversely affected by small geometric offsets of the device during measurement, allowing the delivered dose distributions across linacs to be compared more directly.^20^

#### 2.3.1 Similarity of plan gamma scores

The total percentage of the detectors obtaining a gamma score ≤ 1.0 was recorded using the criteria of (3%,2 mm), (3%,1 mm), and (2%,1 mm), for a global gamma analysis technique and 10% threshold.^20^ This resulted in a plan‐specific gamma score for each plan and linac combination in the study. For each plan, the correlation coefficient was calculated between the MU/cGy modulation factor and the average, minimum and standard deviation of gamma results across all linacs.

#### 2.3.2 Similarity of detector gamma scores and dose difference across linacs

The SNC patient‐calculated gamma scores and dose difference values for each individual detector were exported to MATLAB 2021a (Mathworks, Natick, MA) for further analysis. Throughout this investigation, discussions about a single detector refer to results from individual diodes within the SRSMC, and those about the SRSMC refer to the results given by all diodes combined.

For a given plan, the range and standard deviation of gamma scores and dose difference at each single detector location across all linacs were calculated. This process was then completed for each plan considered in this investigation. There are currently no well‐defined criteria to describe the level of linac matching that one should expect at this level. Routine PSQA criteria were therefore adapted to define metrics for assessment of the similarity between linac deliveries. It was proposed that a fleet of linacs could be considered to deliver matched composite dose distributions if ≥ 95% of detectors displayed a:

- range of gamma ≤ 1.0;
- standard deviation of ≤ 0.5 (∼95% of detectors showing a range of ≤ 1.0).

A further six metrics were proposed based on the global dose differences, with a fleet of linacs considered to deliver matched composite dose distributions if ≥ 95% of detectors displayed a:

- range of dose difference ≤ 3.0% (the most typical dose difference value applied in gamma analysis);
- standard deviation of dose differences ≤ 1.5%;
- range of dose differences ≤ 5.0% (the variation in dose commonly expected to cause noticeable changes in clinical outcome21);
- standard deviation of dose differences ≤ 2.5%;
- range of dose differences ≤ 6.0% (incorporating positive and negative 3% discrepancies)
- standard deviation of dose difference ≤ 3.0%.

These criteria were chosen to consider the similarity of measurements only, not the level of acceptability between the measured data and the TPS. Two results that equally differed from the TPS to an unacceptable level were still concluded to have highly similar deliveries.

#### 2.3.3 Location of detector‐specific gamma fails

The frequency of a detector fail was defined as the number of times a given detector location recorded a failure in the gamma score across all linacs, during the measurement of a given plan. The maximum possible frequency of failure for a given detector and plan combination was therefore equal to the number of investigated linacs.

### 2.4 Evaluation of a distributive patient‐specific quality assurance model

When considering a distributive PSQA model, consisting of the linac on which the patient will be treated (the “treatment linac”) and the linac on which the PSQA will be completed (the “PSQA linac”) there would be four potential outcomes as indicated in Table 3.

**TABLE 3:** Possibly scenarios within a distributive patient‐specific quality assurance (PSQA) model

Treatment linac	PSQA linac	Distributive PSQA outcome
Pass	Pass	True pass
Fail	Fail	True fail
Pass	Fail	False fail
Fail	Pass	False pass

The first two scenarios are desirable and, arguably, a successful implementation of a distributive PSQA model leading to a “True Result.” The third and fourth would lead to failings of a distributive PSQA model. The observed rates of true results, false passes, and false fails that would have been observed across the measurements made in this study, had the clinical plans undergone distributive PSQA were determined at both the composite plan and detector‐specific level.

A PSQA pass result was defined for a detector as a gamma value of ≤ 1.0 and for a plan as 95% of more of the detectors obtaining a pass, using a 10% threshold, regardless of the applied gamma criteria.

#### 2.4.1 Rate of erroneous patient‐specific quality assurance results

Every combination of treatment linac and PSQA linac was considered, giving a total of 1320 combinations. For simplicity, it was assumed that all fractions of a treatment would be delivered on a given linac due to the short treatment courses associated with SBRT.^10^, ^14^

There are currently no well‐defined criteria for the level of matching which should be achieved before implementing a distributive PSQA program. In this investigation, it was proposed that a distributive PSQA program could be considered feasible if the probability of obtaining a false PSQA result were ≤ 5.0%, with no possibility of results being > 5.0% lower than the pass rate defined as PSQA pass criteria.

#### 2.4.2 Rate of erroneous detector gamma result

This process was then repeated at a per‐detector level. All possible combinations of “treatment detector” and “PSQA detector” were considered resulting in a total of 996 406 detector combinations across all plans.

It was proposed that a distributive PSQA program could be considered feasible if less than half of the detectors allowed to fail by the PSQA criteria were expected to differ between PSQA completed on the treatment linac and PSQA linac, for example, 2.5% of detectors when using a 95% pass rate.

## 3 RESULTS

### 3.1 Similarity of deliveries

#### 3.1.1 Similarity of plan gamma scores

**FIGURE 1:**
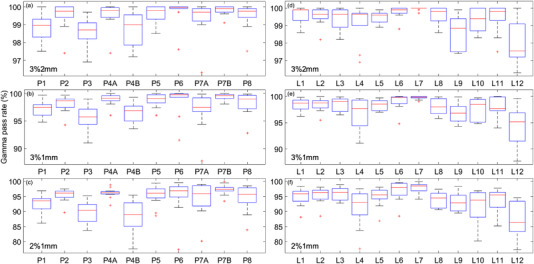
Boxplots of the gamma pass rates across all linacs for each plan (left column) using gamma criterion of: (a) 3%, 2 mm, (b) 3%, 1 mm, (c) 2%, 1 mm, and for each linac across all plans (right column) using a gamma criterion of: (d) 3%, 2 mm, (e) 3%, 1 mm, (f) 2%, 1 mm

The boxplots shown in Figure 1 display the composite plan gamma scores obtained for each linac (L1–L12) when using the criteria of (3%,2 mm), (3%,1 mm), and (2%,1 mm), and similarly the gamma scores per plan. The central marks on the boxplots display the median value, and the edges of each box show the 25th and 75th percentiles (the interquartile range (IQR)). Outliers (red crosses) are identified as points greater than 1.5 times the IQR above or below the boundaries of the IQR.

It can be seen in the boxplots that some linacs produce consistently higher results than others. Of note, at the clinically used criteria of (3%,1 mm), are linac L7 with a minimum score across all plans of 99.0%, and linac L12 with five results of less than 95.0% and a median score of only 95.2%. The median score of plans at criteria of (3%,1 mm), ranged between 95.8% (plan 3) and 99.8% (plan 6). The combined linac and plan dependence can also be observed in the varied IQR values (0.7%–2.8%) across the range of plans and the fleet of linacs.

No correlation was observed between MU/cGy and the average gamma score per plan with correlation coefficients of −0.24, −0.19, and −0.07 for analysis completed at (2%, 1 mm), (3%,1 mm), and (3%,2 mm), respectively. A moderate correlation was observed with the minimum gamma score, correlation coefficients of 0.59, 0.58 and 0.58, and with the standard deviation of gamma scores, correlation coefficients of −0.60, −0.56, and −0.64, for analysis at (2%, 1 mm), (3%,1 mm), and (3%, 2 mm), respectively.

#### 3.1.2 Similarity of detector gamma scores and dose difference across linacs

**FIGURE 2:**
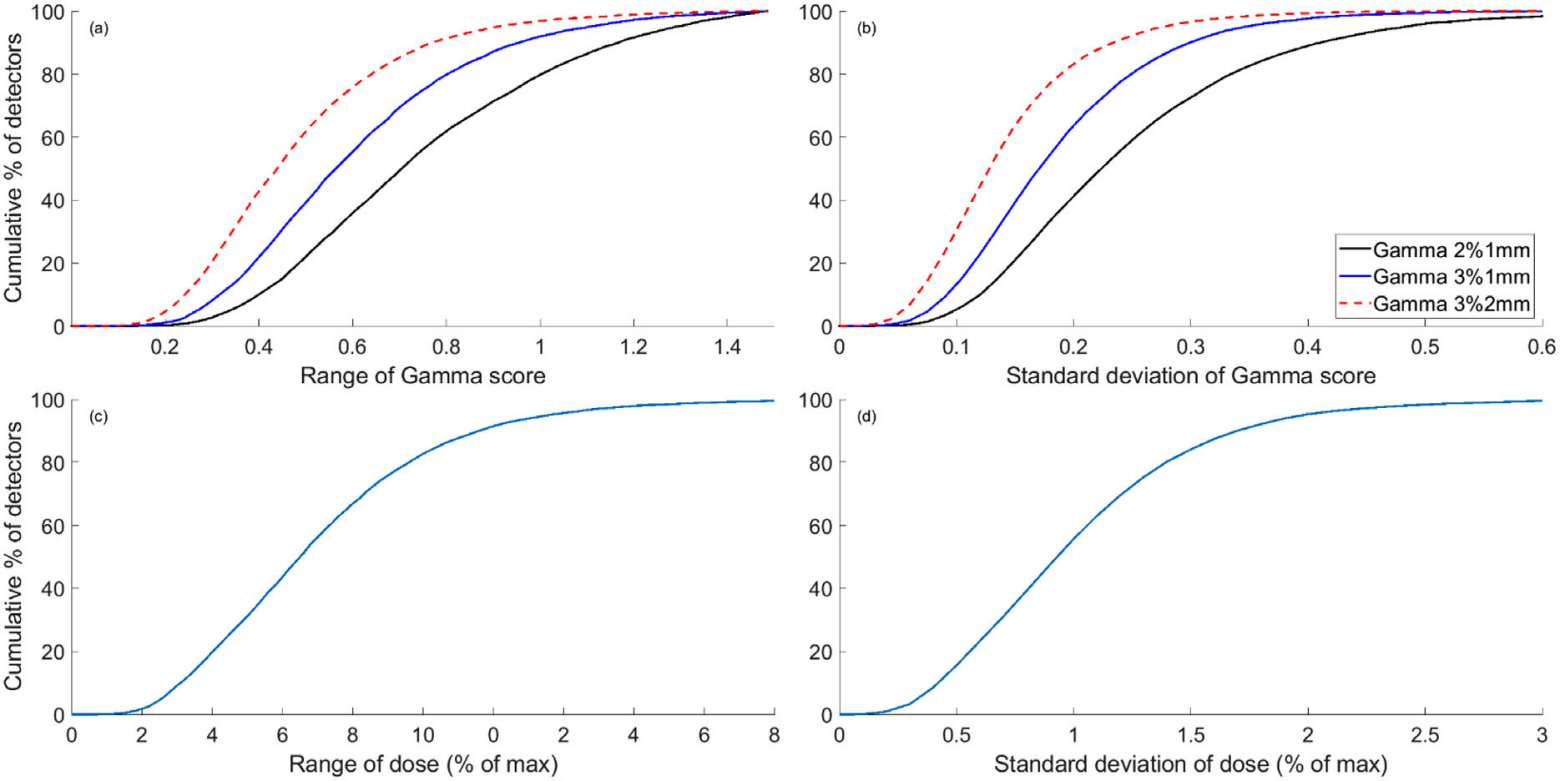
Cumulative histograms displaying: (a) the range of gamma scores, and (b) the standard deviation of gamma scores measured across all linacs, at each detector location, across all plans. Similarly, cumulative histograms of (c) the range of global dose differences, and (d) standard deviation of the global dose differences

The range and standard deviation of gamma scores and global dose differences observed at the same detector location across all measurements are shown in Figure 2. When considering the gamma scores, 80.2%, 92.1%, and 96.9% of detectors showed a range ≤ 1.0 for the criteria of (2%, 1 mm), (3%, 1 mm), and (3%, 2 mm), respectively. Similarly, 95.7%, 99.3%, and 99.9% of detectors displayed a standard deviation of ≤ 0.5 for the criteria of (2%,1 mm), (3%,1 mm), and (3%,2 mm), respectively.

When considering dose differences, 43.6%, 82.7%, and 91.4% of detector measurements showed a range of dose differences ≤ 3.0%, ≤ 5.0%, and ≤ 6.0%, respectively. When considering the standard deviations, 84.1%, 98.3%, and 99.5% of points displayed values of ≤ 1.5%, ≤ 2.5%, and ≤ 3.0%, respectively.

#### 3.1.3 Frequency and location of detector specific gamma fails

**FIGURE 3:**
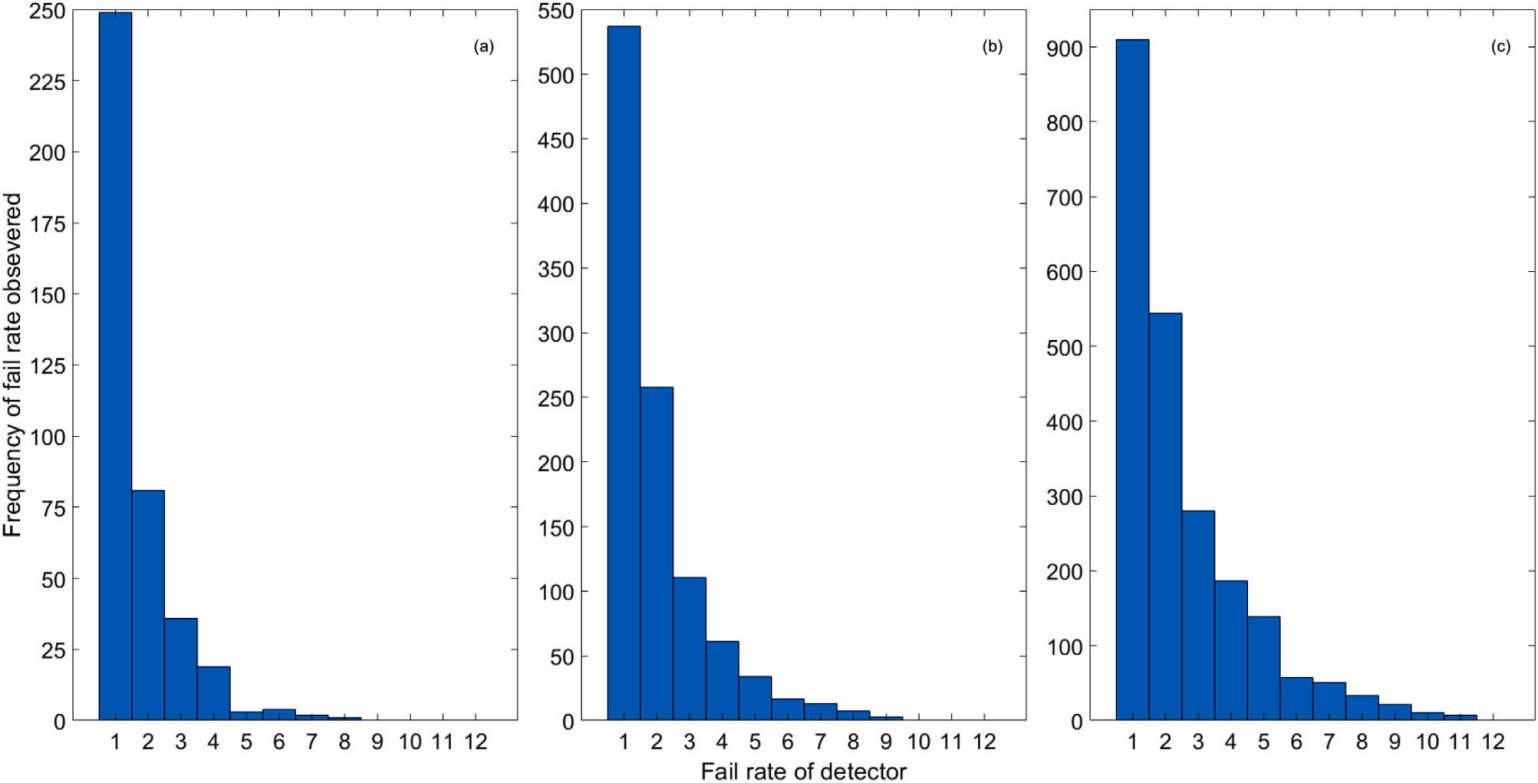
Bar charts showing the frequency of detector fail assessed using gamma criteria of: (a) 3%, 2 mm, (b) 3%, 1 mm, and (c) 2%, 1 mm

The bar charts in Figure 3 show the frequency of detector fails as defined in Section 2.3.3 summated across all plans. If a given detector failed, more than 83.5%, 76.4%, and 64.9% of the time the failure at that given location was observed on only one or two linacs for criteria of (3%, 2 mm), (3%, 1 mm), and (2%, 1 mm), respectively. A given detector was observed to fail for half or more of the linacs only on 0.8%, 2.2%, and 5.4% of occasions for criteria of (3%, 2 mm), (3%, 1 mm), and (2%, 1 mm), respectively.

These results indicate that failing detectors are much more strongly dependent upon the linac used for measurement than the calculation by the TPS. Those detectors which showed a failing score across multiple measurements indicate a strong likelihood that the error is associated with the TPS calculation, rather than the linac delivery, but these are certainly the minority of cases.

**FIGURE 4:**
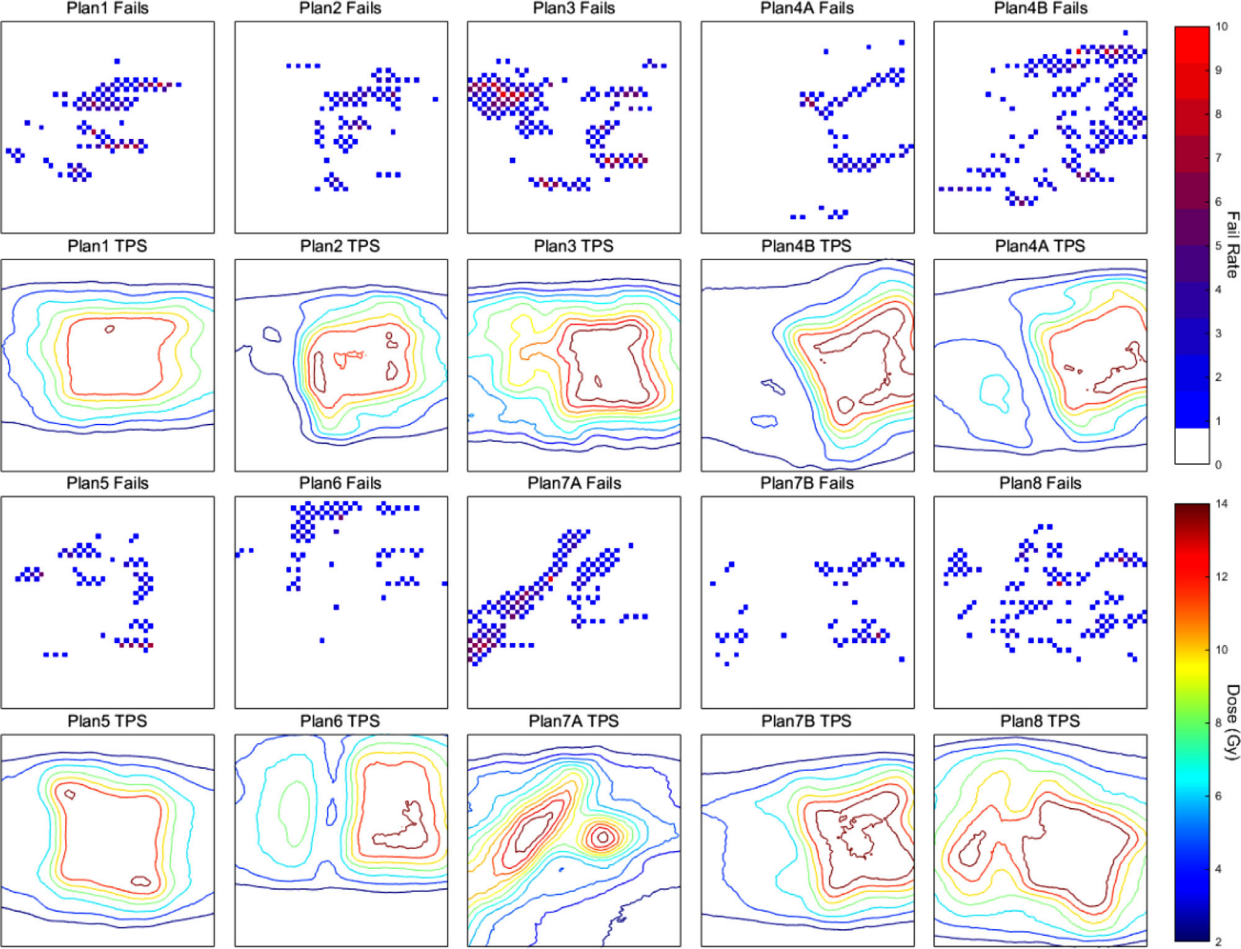
Heat maps of the detector fail locations and the associated isodose distributions from the treatment planning system (TPS). The colorbar associated with the gamma fail maps indicates the number of linacs for which the detector at that location failed the gamma criteria, and the colorbar associated with the isodose maps indicates the expected dose in grey

The distribution of the failure points, across all linacs, obtained during the PSQA measurements can be seen in Figure 4 for a criterion of (3%,1 mm). The plots in the first and third rows show the locations of recorded failures, relative to the calculated isodose distributions shown in the second and fourth rows. As indicated by the bar charts in Figure 3, there were few localized areas of high failure rates, again indicating that the failure points were associated with the specific PSQA measurement and not the TPS calculation.

### 3.2 Evaluation of a distributive patient‐specific quality assurance model

#### 3.2.1 Rate of erroneous patient‐specific quality assurance results

**FIGURE 5:**
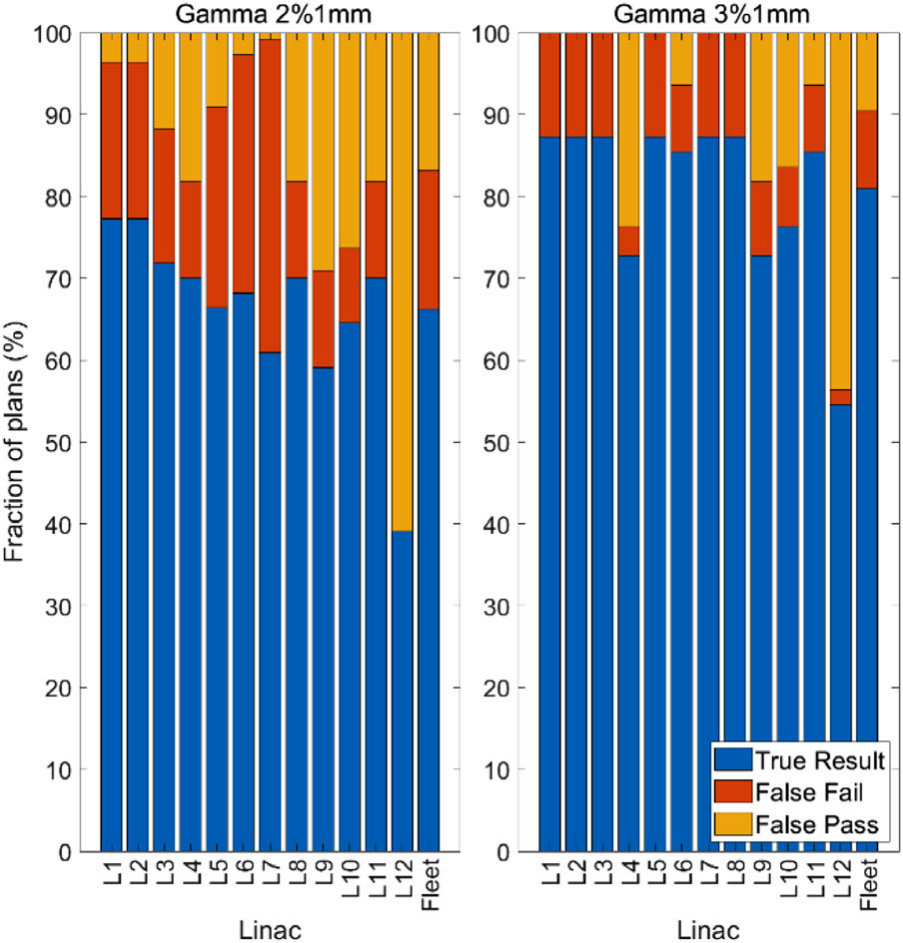
The rate of true result, false pass, and false fail patient‐specific quality assurance (PSQA) results on each linac and the fleet at (2%, 1 mm) (left), and (3% ,1 mm) (right)

The results shown in Figure 5 describe the frequency with which true results, false fails, and false passes would have been observed. When considering the fleet of linacs, a true result would have been obtained for 67.0%, 82.3%, and 100.0% of the possible combinations of treatment linac and PSQA linac for criteria of (2%,1 mm), (3%,1 mm), and (3%,2 mm), respectively.

Some linacs (e.g., linac L12) were more susceptible to false passes which are a concern for patient safety. Other linacs such as L6 and L7 showed a higher susceptibility to false fails as a result of their much higher PSQA results on average. Results for (3%, 2 mm) are not included in Figure 5 as all linacs showed a pass result for all measurements.

#### 3.2.2 Rate of erroneous detector gamma result

**FIGURE 6:**
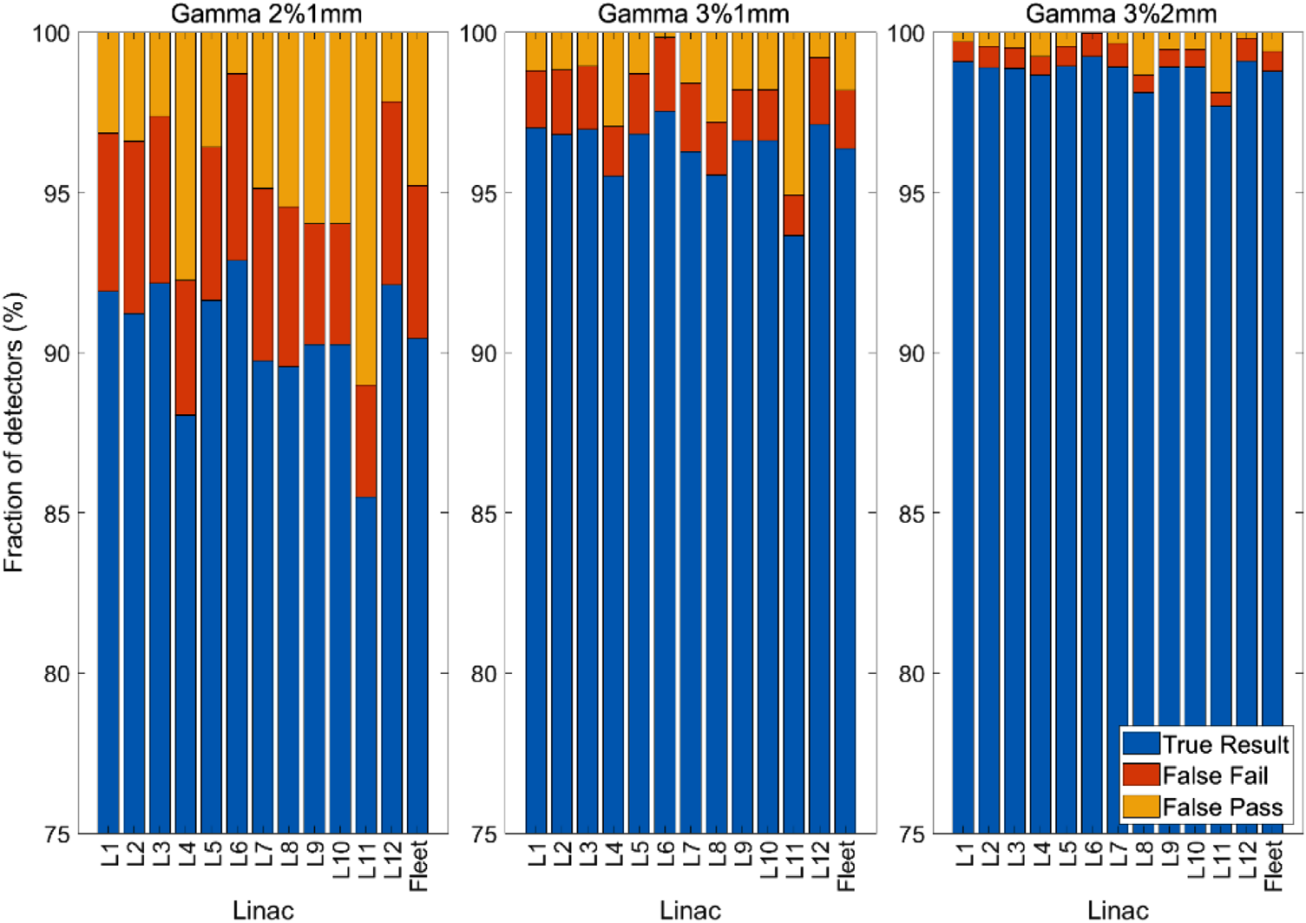
The rate of true results, false passes, and false fails observed at a single detector level for each linac and the fleet at (2%, 1 mm) (left), (3%, 1 mm) (center), and (3%, 2 mm) (right). Note the magnification of the vertical scale relative to Figure 5

The bar charts in Figure 6 show the rate of true result, false passes, and false fails observed at a detector level, assessed using the (2%,1 mm), (3%,1 mm), and (3%,2 mm) criteria.

When considering the fleet of linacs, 90.6%, 96.5%, and 98.8% of detectors displayed true results for criteria of (2%, 1 mm), (3%, 1 mm), and (3%,2 mm), respectively. Although these values are high, it is important to consider the rate of false results against clinical tolerance. During clinical PSQA, common practice is to accept up to 5% of detectors displaying a failing gamma score. As such, one should require the rate of detectors that are expected to display a result different from that observed during the PSQA measurement to be significantly less than 5%.

## 4 DISCUSSION

In this study, metrics have been proposed to determine if the composite dose distributions delivered by a fleet of linacs can be considered matched for SBRT deliveries, and if these PSQA results indicate that a distributive PSQA model could be implemented safely.

### 4.1 Similarity of deliveries

#### 4.1.1 Similarity of plan gamma scores

The variations in the range and standard deviations of gamma scores observed across linacs and plans show that the level of agreement between measurement and the TPS are both linac and plan dependent. This highlights the importance of ensuring an adequate range of linacs and plan types are included in assessments of linac matching to avoid artificially high or low levels of similarity being observed.

Both Rijken et al. and Xu et al. considered linacs to be well matched if the standard deviation of plan gamma results was ≤ 1.0%.^12^, ^13^ This was met for all plans when using the gamma criterion of (3%, 2 mm), for only one plan when using (3%, 1 mm), and for no plans when using (2%, 1 mm). If the worst‐performing two or three linacs were removed from the fleet, the range of gamma results would drastically decrease indicating that improved matching could be achieved for certain linacs.

The observation of moderate correlation between plan modulation and both the minimum and standard deviation of gamma scores indicates that high levels of modulation may be an indicator of the need for PSQA and treatment to be completed in a non‐distributive fashion. From the data gathered here, it could be estimated that acceptable standard deviations in results of less than 1.0% or 1.5%, for a gamma criterion of (3%,1 mm), may be achieved if the plan MU/cGy is maintained < 4.20 or < 5.00, respectively, although more data would be required to conclude this.

#### 4.1.2 Similarity of detector gamma scores and dose difference across linacs

When considering detector level gamma scores, the linacs were concluded to be matched against both proposed metrics when using a gamma criterion of (3%, 2 mm). This supports the finding of the feasibility study performed by Rijken et al.,^12^ but using a much larger dataset. Using a gamma criterion of (3%, 1 mm) or (2%, 1 mm), the fleet could only be considered matched using the less strict metric of ≥ 95% of detectors displaying a standard deviation of ≤ 0.5. These results indicate a very high level of similarity in composite dose deliveries for the best‐performing linacs within the fleet, above that reported by Rijken et al. or Xu et al.^12^, ^13^

When considering the dose differences in place of gamma score, the fleet could not be considered matched against the proposed dose range metrics, highlighting a point of consideration for matched linac departments. Acceptable dose difference criteria are often implemented at ±3% with the aim of ensuring that doses are delivered within ±5% of the TPS calculation.^15^ Linacs delivering at opposing ends of these ranges within a single department, while simultaneously showing acceptable agreement to the TPS, could exceed a difference of 5%, indicated as a level at which a change in clinical outcome can occur.^21^ This implies that differences in clinical outcome could potentially be observed from matched linacs in a department if strict enough criteria are not adhered to.

The proposed metrics in relation to the standard deviation of dose differences were met for ≥ 95% of detectors showing a standard deviation of ≤ 2.5% (and ≤ 3.0%). This further suggests that the observed level of matching is adversely affected by outlier linacs, or measurements points, and provides confidence that any potential variations in clinical outcome would be highly localized.

#### 4.1.3 Location of detector‐specific gamma fails

Figures 3 and 4 present a strong argument for the errors being a result of measurement, either through linac delivery error, or phantom and detector differences, rather than a systematic error within the TPS. This indicates that the differences in delivery are resulted from differences in linac characteristics not directly addressed by the matching criteria proposed by Rijken et al., potentially including the multi‐leaf collimator (MLC) calibration and sags with gantry rotation, MLC speed or the size or shape of the mechanical or radiation isocentres.^12^, ^22^, ^23^, ^24^, ^25^ Although these characteristics were matched at the levels suggested in internationally recognized guidelines,^9^, ^11^, ^26^ stricter tolerances may be required to achieve adequate matching across linacs for SBRT. Alternatively, this could be a result of discrepancies between SRSMC devices; however, most SRSMC devices were used across more than one linac and systematic errors associated with failures at single detector locations for a given SRSMC were not observed.

The use of the SNC “calc shift” increased the similarity of these measurements compared to analysis of the data as measured. During clinical PSQA, it may be desirable to incorporate image guidance to resemble the patient treatment more closely. Daily fusion of patient images carries larger uncertainties than fusions of a phantom however, as such, image‐guided PSQA does not exactly replicate image‐guided treatment. The use of this “calc shift” function, therefore, allowed a more direct comparison of composite delivered doses, without the effects of systematic errors in laser calibration or imaging and treatment isocentre co‐incidence which would be present during distributive treatments.

### 4.2 Evaluation of a distributive patient‐specific quality assurance model

To ensure that the PSQA would yield a passing result on the treatment linac, the PSQA must be completed on that linac. When implementing a distributive PSQA system one must therefore establish a likelihood of a false pass or false failure that is acceptable. This should be considered in combination with the potential severity of the failure that may have been observed if PSQA were completed on the treatment linac and there are treatments for which it may never be deemed suitable.

#### 4.2.1 Rate of erroneous patient‐specific quality assurance results

During this investigation, the frequency of true distributive PSQA results was 100% using a gamma criterion of (3%, 2 mm), and ranged across linacs from 62.7% to 88.2% when using gamma criteria of (3%, 1 mm), and from 43.6% to 78.2% when using a criterion of (2%, 1 mm). The minimum gamma scores were 87.7% (3%, 1 mm) and 77.3% (2%, 1 mm). The metrics proposed in Section 2.4.1 were therefore only met when using a (3%, 2 mm) criterion. For criteria of (3%, 1 mm) or (2%, 1 mm) the risk of a false PSQA result was deemed too high.

#### 4.2.2 Rate of erroneous detector gamma result

Similarly, an acceptable level of false detector results was only observed using a gamma criterion of (3%, 2 mm). Although a high proportion of detectors were observed to give true results for all gamma criteria, typically only 5.0%–10.0% of detectors failing is routinely considered acceptable. The varying locations of the failing detector between measurements also ensure that were it possible to associate the location of failure points with a location in the patient treatment, for example, a PTV or OAR, the information obtained in a distributive PSQA fashion would not be representative of the delivered treatment. This can carry significant risk for SBRT treatments close to an OAR, such as spinal treatments, where fail points in the PTV may be much more acceptable than those in the spinal cord region.

#### 4.2.3 Distributive patient‐specific quality assurance considerations

The metrics proposed in this work are not well established in clinical practice and are subject to further investigation and consideration. There is precedent for reducing the gamma pass tolerance for a plan to 90%, especially when using gamma criteria stricter than (3%, 2 mm).^13^, ^15^ The use of a 90% tolerance would reduce the number of fail results observed to one (from fifteen) when using (3%, 1 mm) and to twenty (from fifty four) when using (2%,1 mm). This could subsequently reduce metrics to consider 90% of detectors, allowing for the distributive QA requirements to be achieved at (3%, 1 mm).

The range of true results, false fails, and false passes differed noticeably among linacs. It may therefore be possible to select a smaller sub‐fleet of linacs for which the proposed metrics could be achieved. If, however, a sufficient process and criteria for matching linacs are not well established, such that all linacs adhering to it allow distributive treatment and PSQA, the criteria should be improved in place of excluded specific linacs from a fleet. This provides increased confidence that matching will be observed for future generated plans and prevents the requirement to run an extremely large number of test cases on any new linac added to the fleet to obtain confidence.

Within this study the variations within the measurements and results are exaggerated by the use of multiple phantoms, detectors, and physicists; however, this accurately reflects a clinically matched linac environment and encompasses uncertainties that do need to be included in the consideration of distributive treatments and PSQA. If moving to a distributive PSQA model, it is advised that a review of the routine linac QA program is completed to ensure clinically acceptable standards are maintained considering a potentially reduced workload in PSQA which can be an early indicator of linac fault. The introduction of a standard plan to be completed using PSQA methodology during monthly linac QA may provide continued confidence in linac performance. All linacs that treat patients in a distributive fashion should complete some quantity of departmental PSQA load to ensure systematic errors do not go undetected.

## 5 CONCLUSION

The present study showed that a fleet of 12 matched Elekta linacs were able to deliver spinal SBRT composite dose distributions with a high level of similarity when assessed using a gamma criterion of (3%, 2 mm). The standard deviation of gamma scores for each of the 10 plans was less than 1.0% and ≥ 95% of detectors showed a range of gamma scores ≤1.0. When reducing the gamma criteria, the similarity of composite measurement was reduced, but the majority maintained a high level of agreement with ≥ 95% of detectors showing a standard deviation of gamma scores ≤1.0. Matching linacs to the criteria suggested by Rijken et al., therefore, resulted in highly similar complex dose distribution delivery.

The locations of failure points observed were shown to be inconsistent across measurements. Discrepancies from the TPS calculated doses were therefore due to linac performance or measurement, and not systematic TPS errors. To obtain more consistent PSQA results, the focus should be on increasing the matching of the linac performance.

The level of similarity observed would allow patient treatments to be delivered, and PSQA to be completed in a distributive fashion, if a tolerance of 95% of points for a criterion of (3%, 2 mm) was deemed adequate for clinical practice. When using the current GenesisCare tolerance of 95% of points and gamma criterion of (3%,1 mm), the proposed distributive PSQA metrics were not achieved. It was therefore recommended that PSQA be completed on a single linac which is subsequently used for all fractions of a patient treatment until improved matching can be achieved.

## AUTHOR CONTRIBUTION

Simon Goodall is the guarantor of integrity of the entire study. Simon Goodall, Leon Dunn, Luis Munoz and Jonathan Dunning substantially contributed to data acquisition during this study. All authors provided input to the design of the study, critical revision of data analysis and review of the manuscript and figures.

## CONFLICT OF INTEREST

The authors declare that there is no conflict of interest that could be perceived as prejudicing the impartiality of the research reported.
